# Correction: Civil war and death in Yemen: Analysis of SMART survey and ACLED data, 2012–2019

**DOI:** 10.1371/journal.pgph.0001915

**Published:** 2023-05-03

**Authors:** Debarati Guha Sapir, Jideofor Thomas Ogbu, Sarah Elizabeth Scales, Moitinho de Almeida, Akram al-Masnai, Anne-Francoise Donneau, Anh Diep, Robyn Bernstein, Jose Manuel Rodriguez-Llanes, Gilbert Burnham

The figures were published out of order. See here the correct figures and their captions.

**Fig 1:**
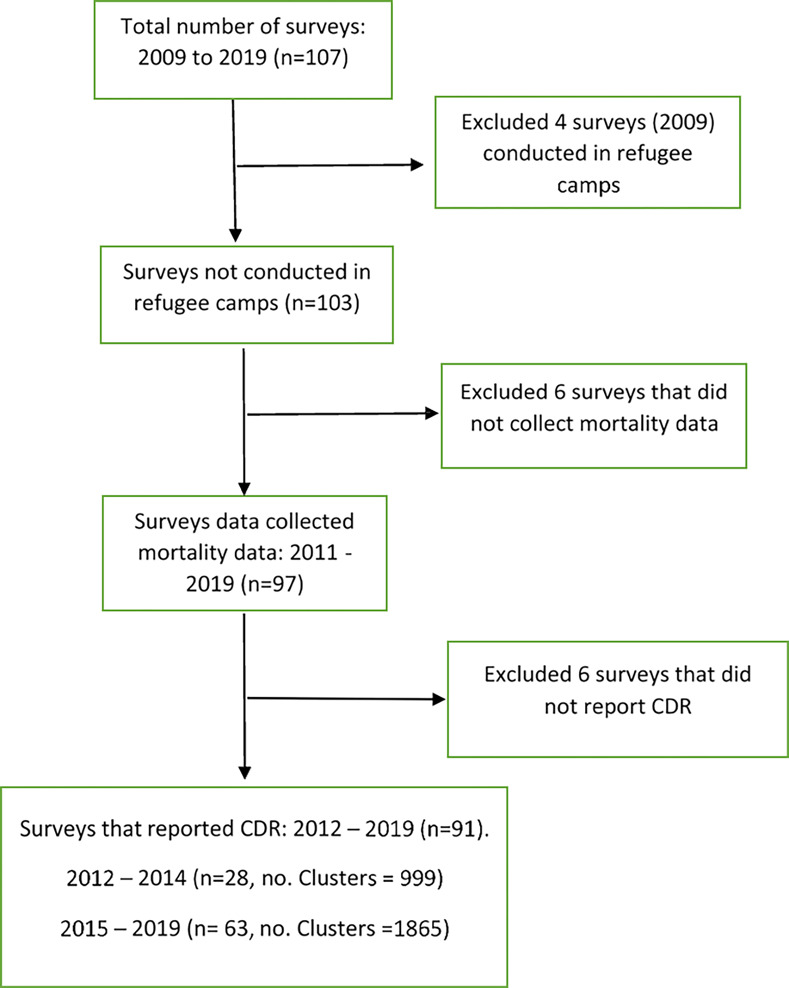
Survey selection flowchart, describing the survey selection process, inclusion/exclusion criteria, and number of surveys at each selection stage.

**Fig 2:**
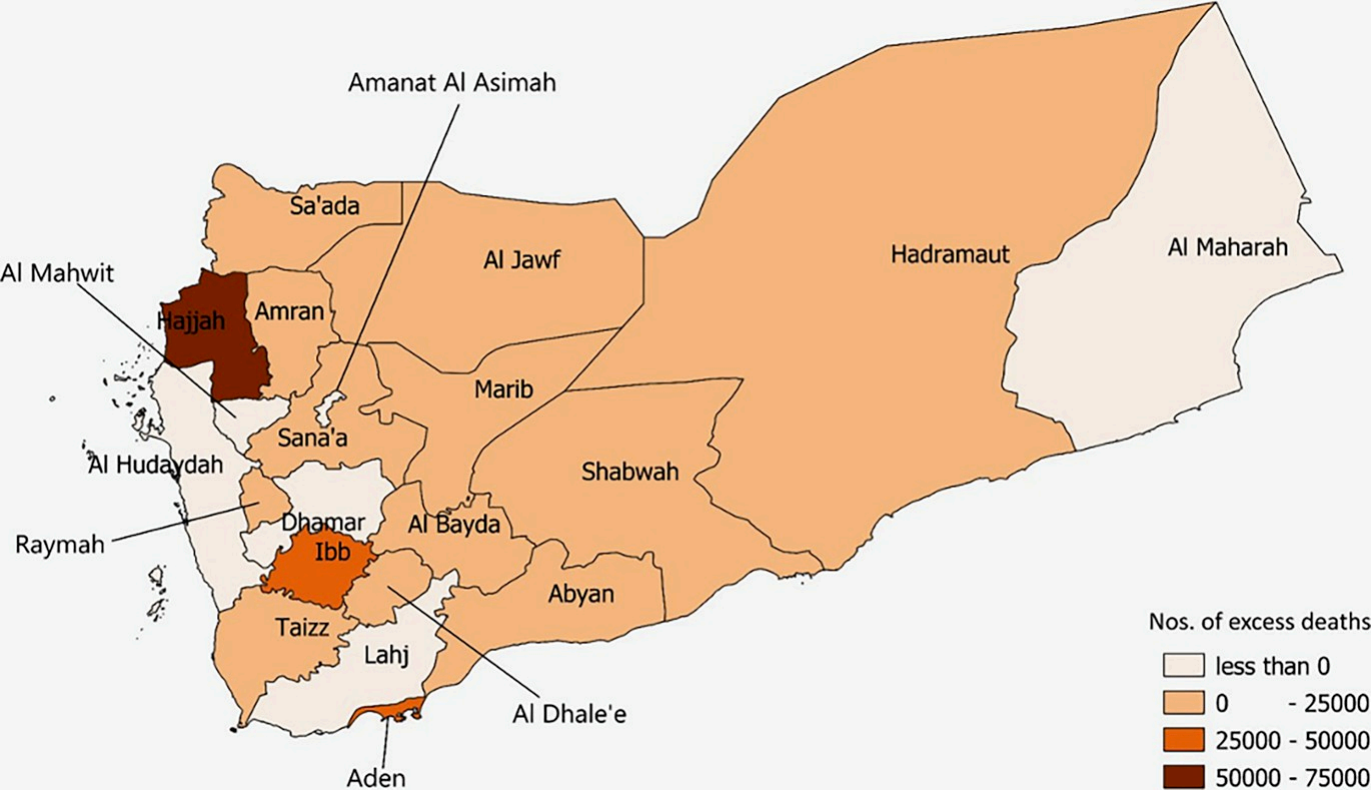
Geographical distribution of total excess deaths based on excess death rates by governorate, Yemen, 2015–2019. Source: Shape files extracted from Global Administrative Areas (2012). GADM database of Global Administrative Areas, version 2.0. [online] URL: www.gadm.org. Created using QGIS version 3.10.3.

**Fig 3:**
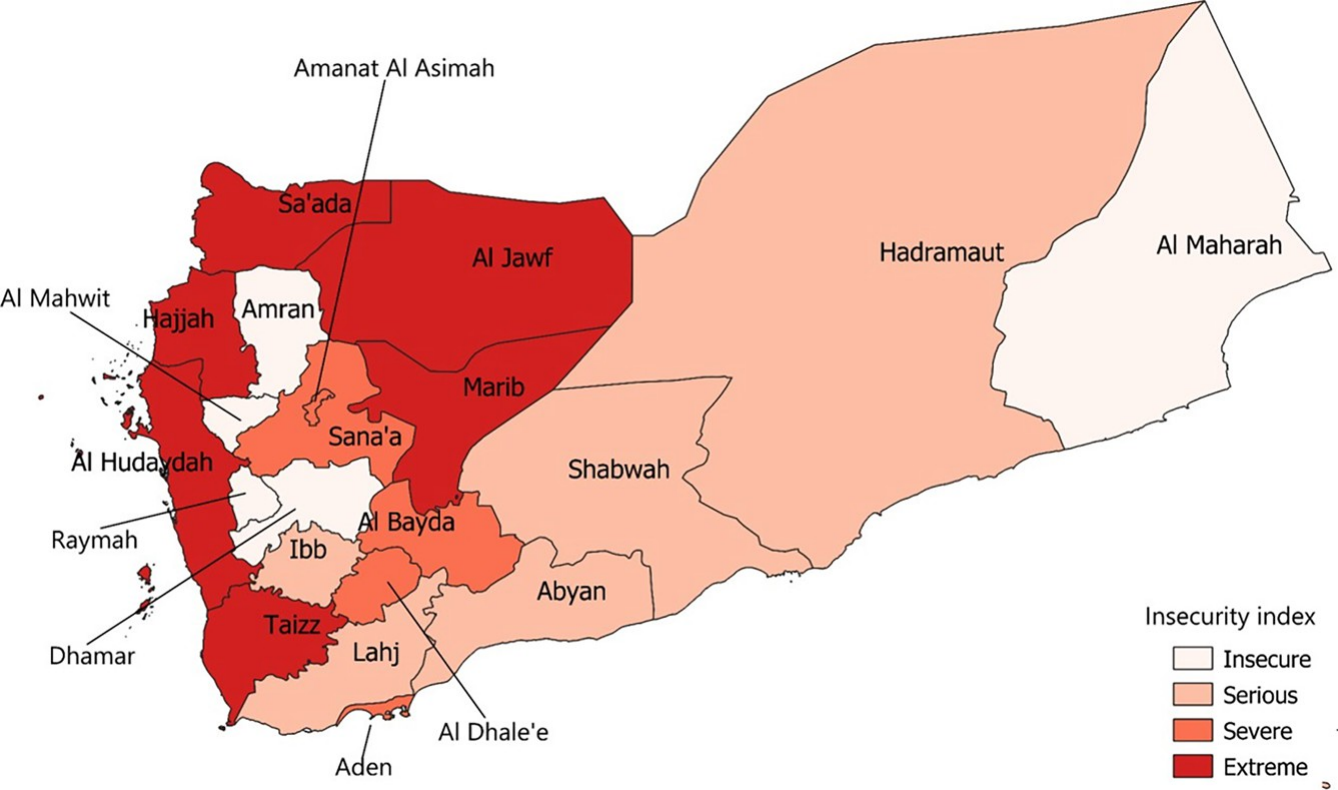
Geographic distribution of insecurity index levels, Yemen, 2015–2019.

**Fig 4:**
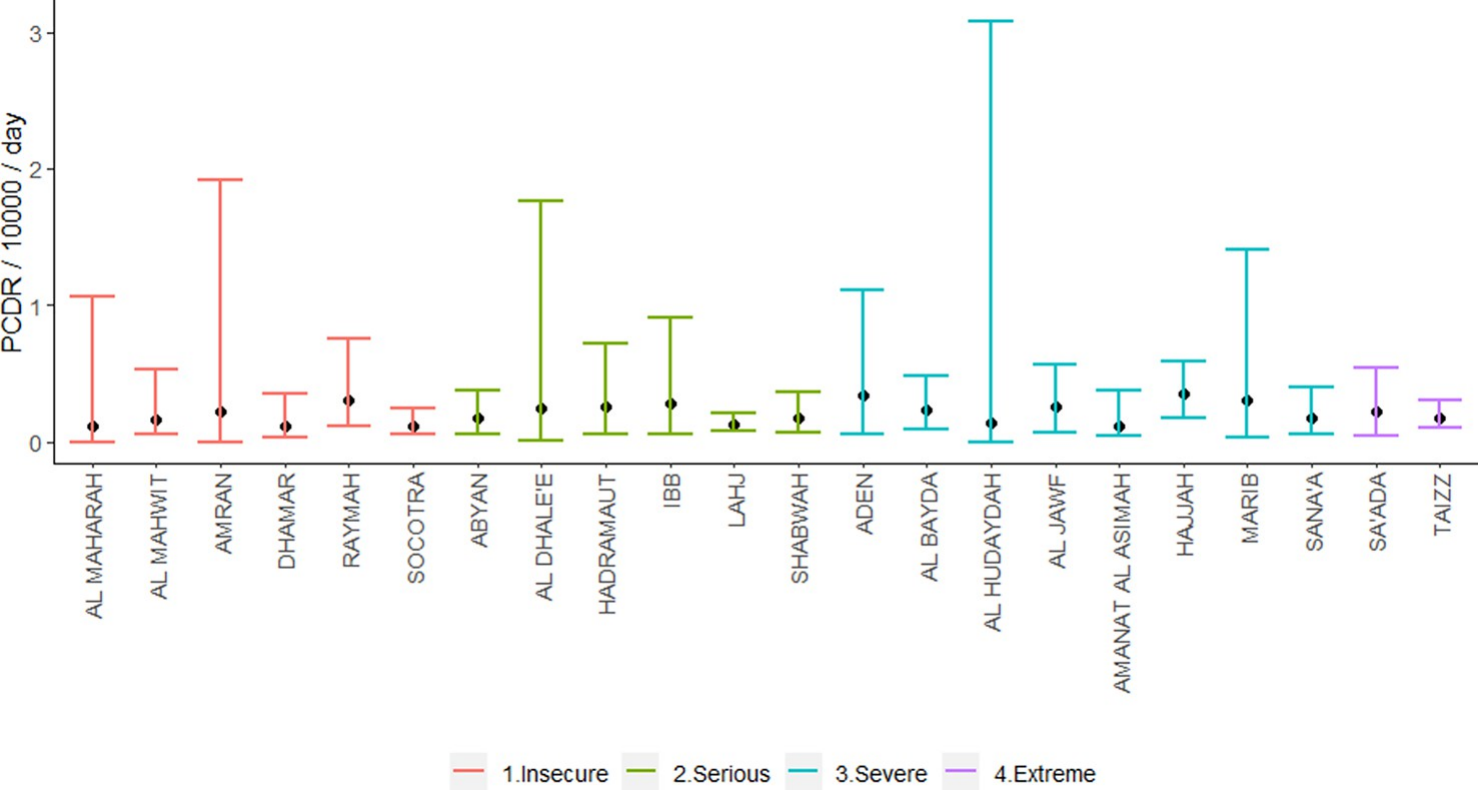
Posterior crude death rate by governorate and insecurity level, Yemen, 2015–2019.

**Fig 5:**
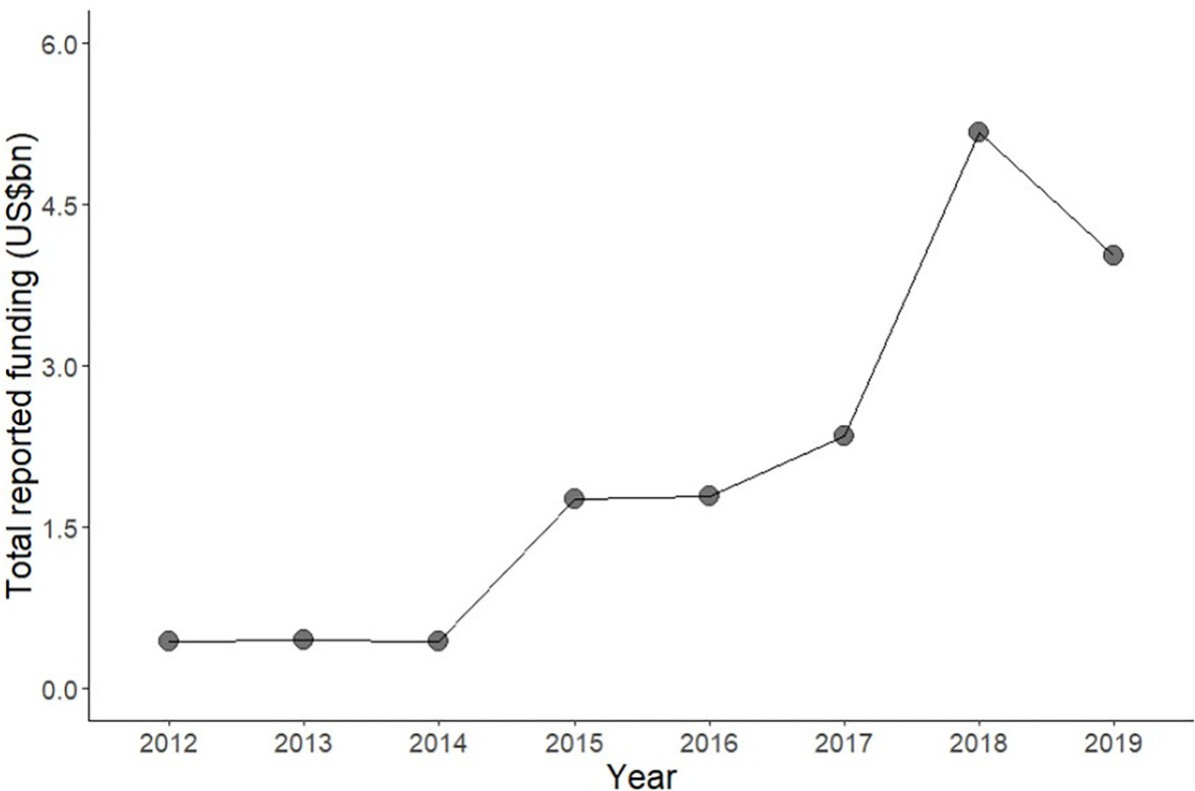
Humanitarian aid assistance to Yemen 2012–2019. Data source: United Nations Office for the Coordination of Humanitarian Aid (UN-OCHA) [52].
