# Supplementary figures and images for: Civil war and death in Yemen: Analysis of SMART survey and ACLED data, 2012–2019
Source: PLOS Glob Public Health. 2022 Aug 8;2(8):e0000581. doi: 10.1371/journal.pgph.0000581 (PMC10022117; doi:10.1371/journal.pgph.0000581)

**S1 Figure: Step-by-step explanation of the calculation of excess deaths in Yemen 2015 - 2019**

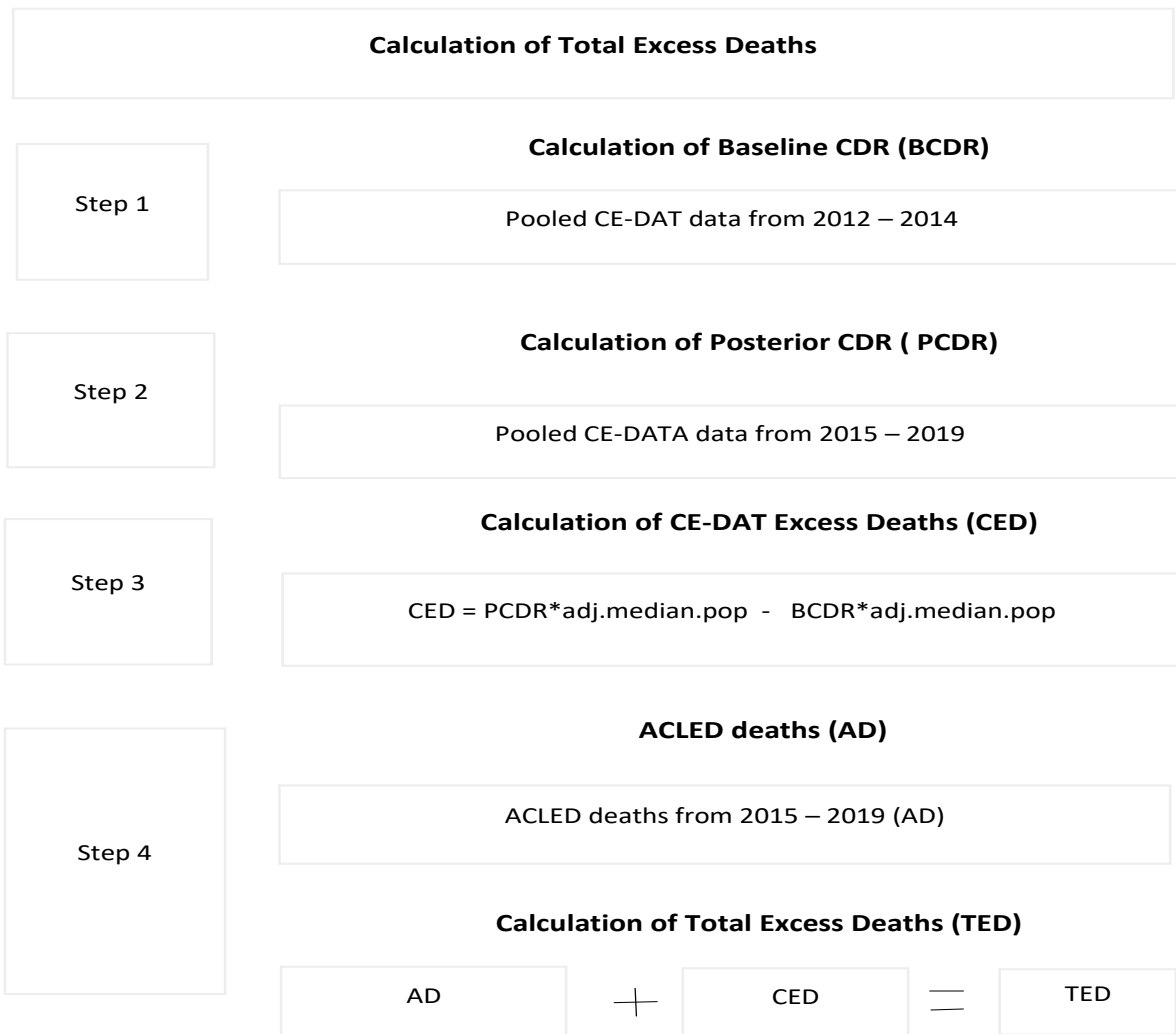

Supplement: S1 Fig — (PDF) [file pgph.0000581.s001.pdf]
