# Supplementary material for: Civil war and death in Yemen: Analysis of SMART survey and ACLED data, 2012–2019
Source: PLOS Glob Public Health. 2022 Aug 8;2(8):e0000581. doi: 10.1371/journal.pgph.0000581 (PMC10022117; doi:10.1371/journal.pgph.0000581)
Supplement: S1 Table — The map of controlled area has been sourced from European Council on Foreign Relations website and has been adapted to include number of air raid/drone strikes. It should be considered indicative only and the page can be accessed by the link below: https://ecfr.eu/publication/talking_to_the_houthis_how_europeans_can_promote_peace_in_yemen/. (PDF) [file pgph.0000581.s002.pdf]

S1 Table: Distribution of air raids/drone attacks, associated direct deaths, and PCDR by Governorate, Yemen, Jan 2015 – Dec 2019.

Governorates	Direct deaths[b]	Air and drone attacks[b]	PCDR [c]	Level of insecurity
Socotra[a]	0	0	0.12	1
Al Mahrah	0	1	0.12	1
Raymah	7	15	0.30	1
Hadramawt	169	43	0.26	2
Al Mahwit	27	65	0.16	1
Abyan	332	117	0.18	2
Dhamar	371	161	0.11	1
Ad Dali	464	166	0.25	2
Aden	325	213	0.34	3
Ibb	313	214	0.28	2
Lahij	420	317	0.13	2
Shabwah	507	319	0.17	2
Amran	359	378	0.22	1
Al Bayda	781	452	0.23	3
Al Jawf	660	752	0.26	3
Marib	885	1323	0.30	3
Amanat al A.	1197	1325	0.12	3
Al Hodeida	2639	1755	0.14	3
Hajjah	2176	1945	0.35	3
Taizz	2549	1978	0.18	4
Sana'a	979	2013	0.17	3
Sa'dah	3520	4932	0.22	4

[a] Socotra is a group of four sparsely populated islands forming an archipelago located about 340 km off the coast of Yemen.

[b] Source: extracted from ACLED /Yemen Data Project

[c] CDR calculated from our model
