# Supplementary material for: Civil war and death in Yemen: Analysis of SMART survey and ACLED data, 2012–2019
Source: PLOS Glob Public Health. 2022 Aug 8;2(8):e0000581. doi: 10.1371/journal.pgph.0000581 (PMC10022117; doi:10.1371/journal.pgph.0000581)
Supplement: S2 Table — Characteristics of 91 small-scale mortality surveys in Yemen, 2012–2019. All surveys were reviewed for mortality estimates. This information and more resources can be accessed at: https://www.humanitarianresponse.info/en/operations/yemen/nutrition. (PDF) [file pgph.0000581.s003.pdf]

S2 Table: Surveys characteristics, Yemen, 2015 – 2019. *Characteristics of 91 small-scale mortality surveys in Yemen, 2012-2019. All surveys were reviewed for mortality estimates.*

S.ID	GOVERNORATE	TITLE	CLUSTER	YEAR	REPRESENTATIVE SAMPLE SIZE
1A	ADEN	NUTRITIONAL SURVEY REPORT ADEN GOVERNORATE		2012	10442
2A	HAJJAH	LOWLAND AND MOUNTAINOUS ECOLOGICAL ZONES HAJJAH GOVERNORATE	HL	2012	4516
2B	HAJJAH	LOWLAND AND MOUNTAINOUS ECOLOGICAL ZONES HAJJAH GOVERNORATE	LL	2012	4948
3A	HAJJAH	NUTRITION SURVEY REPORT SETTLEMENTS OF INTERNALLY DISPLACED PERSONS HAJJAH	IDP	2012	5175
4A	IBB	NUTRITIONAL SURVEY EASTERN AND WESTERN HIGH LAND IBB	EHL	2012	8592
4B	IBB	NUTRITIONAL SURVEY EASTERN AND WESTERN HIGH LAND IBB	WHL	2012	10032
5A	LAHEG	NUTRITION AND MORTALITY SURVEY IN LOW LAND AND MOUNTAINOUS ECOLOGICAL ZONES OF LAHJ GOVERNORATE YEMEN	HL	2012	4970
5B	LAHEG	NUTRITION AND MORTALITY SURVEY IN LOW LAND AND MOUNTAINOUS ECOLOGICAL ZONES OF LAHJ GOVERNORATE YEMEN	LL	2012	5489

6A	REYMAH	NUTRITION SURVEY OF RAYMA GOVERNORATE		2012	576700
7A	TAIZ	MOUNTAINOUS AND COASTAL PLAIN ECOLOGICAL ZONES TAIZ GOVERNORATE	TM	2012	4100
7B	TAIZ	MOUNTAINOUS AND COASTAL PLAIN ECOLOGICAL ZONES TAIZ GOVERNORATE	TC	2012	4827
8A	ABYAN	CONFLICT DIRECTLY AFFECTED AND INDIRECTLY AFFECTED DISTRICTS ABYAN GOVERNORATE	CIDA	2013	8344
8B	ABYAN	CONFLICT DIRECTLY AFFECTED AND INDIRECTLY AFFECTED DISTRICTS ABYAN GOVERNORATE	CDA	2013	9176
9A	AL-MAHWEET	NUTRITIONAL SURVEY AL MAHWEET GOVERNORATE YEMEN: HIGHLANDS AND LOWLANDS	HL	2013	5854
9B	AL-MAHWEET	NUTRITIONAL SURVEY AL MAHWEET GOVERNORATE YEMEN: HIGHLANDS & LOWLANDS DISTRICTS	LL	2013	6831
10A	DHAMAR	NUTRITIONAL SURBEY DHAMAR EASTERN AND WESTERN DISTRICTS	EASTERN	2013	6442
10B	DHAMAR	NUTRITIONAL SURBEY DHAMAR EASTERN AND WESTERN DISTRICTS	WESTERN	2013	6916

11A	AL-HODEIDAH	MINISTRY OF PUBLIC HEALTH AND POPULATION & UNITED NATIONS CHILDREN'S FUND SMART NUTRITION SURVEY REPORT, HODEIDAH GOVERNORATE 2014, YEMEN	HL	2014	3881
11B	AL-HODEIDAH	MINISTRY OF PUBLIC HEALTH AND POPULATION & UNITED NATIONS CHILDREN'S FUND SMART NUTRITION SURVEY REPORT, HODEIDAH GOVERNORATE 2014, YEMEN	LL	2014	4905
12A	HAJJAH	NUTRITION ANTHROPOMETRIC AND RETROSPECTIVE MORTALITY SURVEY CHILDREN 6 TO 59 MONTHS: LOWLAND AND MOUNTAINOUS ECOLOGICAL ZONES HAJJA GOVERNORATES	LL	2014	4751
12B	HAJJAH	NUTRITION ANTHROPOMETRIC AND RETROSPECTIVE MORTALITY SURVEY CHILDREN 6 TO 59 MONTHS: LOWLAND AND MOUNTAINOUS ECOLOGICAL ZONES HAJJA GOVERNORATES	HL	2014	4631
13A	LAHEG	NUTRITIONAL ANTHROPOMETRIC AND RETROSPECTIVE MORTALITY SURVEY CHILDREN 6 TO 59 MONTHS LOWLAND AND MOUNTAINOUS ECOLOGICAL ZONES LAHJ GOVERNORATE	HL	2014	5489
13B	LAHEG	NUTRITIONAL ANTHROPOMETRIC AND RETROSPECTIVE MORTALITY SURVEY CHILDREN 6 TO 59 MONTHS LOWLAND AND MOUNTAINOUS ECOLOGICAL ZONES LAHJ GOVERNORATE	LL	2014	4516

14A	SAADAH	NUTRITION ANTHROPOMETRIC AND RETROSPECTIVE MORTALITY SURVEY CHILDREN 6 TO 59 MONTHS: LOWLAND AND MOUNTAINOUS ECOLOGICAL ZONES SAADA GOVERNORATES	LL	2014	5072
14B	SAADAH	NUTRITION ANTHROPOMETRIC AND RETROSPECTIVE MORTALITY SURVEY CHILDREN 6 TO 59 MONTHS: LOWLAND AND MOUNTAINOUS ECOLOGICAL ZONES SAADA GOVERNORATES	HL	2014	4596
15A	TAIZ	NUTRITIONAL ANTHROPOMETRIC AND RETROSPECTIVE MORTALITY SURVEY CHILDREN 6 TO 59 MONTHS TAIZ DISTRICT; MOUNTAINOUS, HILLS & VALLEYS AND COASTAL PLAIN ECOLOGICAL ZONES	LL	2014	3468
15B	TAIZ	NUTRITIONAL ANTHROPOMETRIC AND RETROSPECTIVE MORTALITY SURVEY CHILDREN 6 TO 59 MONTHS TAIZ DISTRICT; MOUNTAINOUS, HILLS & VALLEYS AND COASTAL PLAIN ECOLOGICAL ZONES	MZ	2014	3545
15C	TAIZ	NUTRITIONAL ANTHROPOMETRIC AND RETROSPECTIVE MORTALITY SURVEY CHILDREN 6 TO 59 MONTHS TAIZ DISTRICT; MOUNTAINOUS, HILLS & VALLEYS AND COASTAL PLAIN ECOLOGICAL ZONES	MT	2014	4137
16A	ADEN	NUTRITION AND MORTALITY SURVEY ADEN GOVERNORATE		2015	2994
17A	AL-BAIDA	NUTRITION AND MORTALITY SURVEY IN AL-BAIDHA GOVERNORATE		2015	3575
18A	AL-HODEIDAH	NUTRITION AND MORTALITY SURVEY IN HODEIDAH LOWLAND	LL	2015	3738

19A	HAJJAH	NUTRITION AND RETROSPECTIVE MORTALITY SURVEY HIGHLANDS AND LOWLANDS LIVELIHOOD ZONES OF HAJJAH GOVERNORATE	MT	2015	3860
19B	HAJJAH	NUTRITION AND RETROSPECTIVE MORTALITY SURVEY HIGHLANDS AND LOWLANDS LIVELIHOOD ZONES OF HAJJAH GOVERNORATE	LL	2015	3441
20A	LAHEG	NUTRITION AND MORTALITY SURVEY IN LOWLAND AND HIGHLANDS ECOLOGICALZONES LAHJ	HL	2015	2809
20B	LAHEG	NUTRITION AND MORTALITY SURVEY IN LOWLAND AND HIGHLANDS ECOLOGICALZONES LAHJ	LL	2015	3877
21A	ABYAN	EMERGENCY FOOD SECURITY AND NUTRITION ASSESSMENT, YEMEN		2016	631400
21B	ADEN	EMERGENCY FOOD SECURITY AND NUTRITION ASSESSMENT, YEMEN		2016	890100
22A	AL DHALE	NUTRITION SURVEY REPORT AL DHALE GOVERNORATE, YEMEN		2016	4754
21C	AL DHALE	EMERGENCY FOOD SECURITY AND NUTRITION ASSESSMENT, YEMEN		2016	688800
21D	AL JAWF	EMERGENCY FOOD SECURITY AND NUTRITION ASSESSMENT, YEMEN		2016	680400
21E	AL-BAIDA	EMERGENCY FOOD SECURITY AND NUTRITION ASSESSMENT, YEMEN		2016	780200
21F	AL-HODEIDAH	EMERGENCY FOOD SECURITY AND NUTRITION ASSESSMENT, YEMEN		2016	3520800
21G	AL-MAHWEET	EMERGENCY FOOD SECURITY AND NUTRITION ASSESSMENT, YEMEN		2016	795400
21H	AMANAT-AL-ASIMAH	EMERGENCY FOOD SECURITY AND NUTRITION ASSESSMENT, YEMEN		2016	2606400

21I	AMRAN	EMERGENCY FOOD SECURITY AND NUTRITION ASSESSMENT, YEMEN		2016	1251600
21J	DHAMAR	EMERGENCY FOOD SECURITY AND NUTRITION ASSESSMENT, YEMEN		2016	2244600
21K	HADHRAMOUT	EMERGENCY FOOD SECURITY AND NUTRITION ASSESSMENT, YEMEN		2016	1795210
21L	HAJJAH	EMERGENCY FOOD SECURITY AND NUTRITION ASSESSMENT, YEMEN		2016	2140900
21M	IBB	EMERGENCY FOOD SECURITY AND NUTRITION ASSESSMENT, YEMEN		2016	3333800
21N	LAHEG	EMERGENCY FOOD SECURITY AND NUTRITION ASSESSMENT, YEMEN		2016	1095007
21O	MAREB	EMERGENCY FOOD SECURITY AND NUTRITION ASSESSMENT, YEMEN		2016	351000
21P	REYMAH	EMERGENCY FOOD SECURITY AND NUTRITION ASSESSMENT, YEMEN		2016	576700
23A	SAADAH	NUTRITION SURVEY OF SA’ADAH GOVERNORATE	SHL	2016	3793
23B	SAADAH	NUTRITION SURVEY OF SA’ADAH GOVERNORATE	SLL	2016	3817
24A	SANAA	NUTRITION SURVEY OF SANA'A GOVERNORATE	SAD	2016	3448
24B	SANAA	NUTRITION SURVEY OF SANA'A GOVERNORATE	SAT	2016	4070
21Q	SANAA	EMERGENCY FOOD SECURITY AND NUTRITION ASSESSMENT, YEMEN		2016	1385500
21R	SHABWA	EMERGENCY FOOD SECURITY AND NUTRITION ASSESSMENT, YEMEN		2016	85840
25A	TAIZ	NUTRITION SURVEY OF TAIZ GOVERNORATE	TCITY	2016	2605
25B	TAIZ	NUTRITION SURVEY OF TAIZ GOVERNORATE	THL	2016	3572

25C	TAIZ	NUTRITION SURVEY OF TAIZ GOVERNORATE	TLL	2016	3371
26A	IBB	NUTRITION AND MORTALITY SURVEY REPORT IBB GOVERNORATE, YEMEN	WHL	2017	10032
26B	IBB	NUTRITION AND MORTALITY SURVEY REPORT IBB GOVERNORATE, YEMEN	EHL	2017	8592
27A	SHABWA	NUTRITION AND MORTALITY SURVEY REPORT SHABWA GOVERNORATE, YEMEN	Plateau	2017	4102
27B	SHABWA	NUTRITION AND MORTALITY SURVEY REPORT SHABWA GOVERNORATE, YEMEN	Lowland Coastal	2017	4246
28A	TAIZ	EMERGENCY WASH & NUTRITION FOR CONFLICT AFFECTED PEOPLE IN YEMEN - TAIZ GOVERNORATE	HL	2017	3884
29A	ABYAN	NUTRITION AND RETROSPECTIVE MORTALITY SURVEY HIGHLANDS AND LOWLANDS LIVELIHOOD ZONES OF ABYAN GOVERNORATE	LL	2018	3501
29B	ABYAN	NUTRITION AND RETROSPECTIVE MORTALITY SURVEY HIGHLANDS AND LOWLANDS LIVELIHOOD ZONES OF ABYAN GOVERNORATE	HL	2018	3650
30A	ADEN	SMART SURVEY PRELIMINARY RESULT, ADEN GOVERNORATE, YEMEN		2018	4613
31A	AL DHALE	SMART SURVEY PRELIMINARY RESULT, AL DHALE GOVERNORATE, YEMEN		2018	4754

32A	AL JAWF	REPORT ON THE NUTRITIONAL STATUS AND MORTALITY SURVEY, AL JAWF GOVERNORATE, YEMEN		2018	4426
33A	AL-BAIDA	NUTRITIONAL STATUS AND MORTALITY REPORT AL-BAIDA GOVERNORATE, YEMEN		2018	3576
34A	AL-MAHRA	SMART SURVEY PRELIMINARY RESULT, AL-MAHRA GOVERNORATE, YEMEN		2018	5906
35A	AMRAN	NUTRITIONAL STATUS AND MORTALITY REPORT AMRAN GOVERNORATE, YEMEN		2018	4537
36A	HADHRAMOUT	SMART SURVEY PRELIMINARY RESULT, COASTAL ZONE, AND VALLEY & DESERT ZONE, HADHRAMOUT GOVERNORATE, YEMEN	CZ	2018	4929
36B	HADHRAMOUT	SMART SURVEY COASTAL ZONE, AND VALLEY & DESERT ZONE, HADHRAMOUT GOVERNORATE	VDZ	2018	5691
37A	HAJJAH	NUTRITION AND MORTALITY SURVEY HIGHLANDS AND LOWLANDS LIVELIHOOD ZONES OF HAJJAH	LL	2018	3373
37B	HAJJAH	NUTRITION AND RETROSPECTIVE MORTALITY SURVEY HIGHLANDS AND LOWLANDS LIVELIHOOD ZONES OF HAJJAH GOVERNORATE	HL	2018	4663
38A	LAHEG	SMART SURVEY PRELIMINARY RESULT, HIGH LAND, AND LOWLAND ZONE, LAHEG GOVERNORATE,	HL	2018	3521
38B	LAHEG	SMART SURVEY PRELIMINARY RESULT, HIGH LAND, AND LOWLAND ZONE, LAHEG GOVERNORATE,	LL	2018	3031

39A	MAREB	REPORT ON THE NUTRITIONAL STATUS AND MORTALITY SURVEY, MAREB GOVERNORATE, 2018	RURAL	2018	3900
39B	MAREB	REPORT ON THE NUTRITIONAL STATUS AND MORTALITY SURVEY, MAREB GOVERNORATE, 2018	CITY	2018	3373
40A	SANAA	SMART SURVEY REPORT FOR NUTRITIONAL STATUS AND MORTALITY SANAA GOVERNORATE, Y	DZ	2018	3984
40B	SANAA	SMART SURVEY REPORT FOR NUTRITIONAL STATUS AND MORTALITY SANAA GOVERNORATE,	TZ	2018	3278
41A	SOCOTRA	NUTRITION AND MORTALITY SURVEY REPORT SOCOTRA GOVERNORATE, YEMEN		2018	4808
42A	TAIZ	NUTRITION, MORTALITY AND FOOD SECURITY SURVEY FOLLOWING THE SMART SURVEY METHODOLOGY IN TAIZ GOVERNORATE YEMEN	TLL	2018	2820
42B	TAIZ	NUTRITION, MORTALITY AND FOOD SECURITY SURVEY FOLLOWING THE SMART SURVEY	THL	2018	2973
42C	TAIZ	NUTRITION, MORTALITY AND FOOD SECURITY SURVEY FOLLOWING THE SMART SURVEY METHODOLOGY IN TAIZ GOVERNORATE YEMEN	TC	2018	3835
43A	AL-MAHRA	SMART SURVEY PRELIMINARY RESULT, AL-MAHRA GOVERNORATE, YEMEN		2019	3017
43B	SHABWA	SMART SURVEY PRELIMINARY RESULT, SHABWA GOVERNORATE, YEMEN		2019	4797
44A	SOCOTRA	SMART SURVEY PRELIMINARY RESULT, SOCOTRA GOVERNORATE, YEMEN		2019	2961

This information and more resources can be accessed at: *https://www.humanitarianresponse.info/en/operations/yemen/nutrition*.
