# Supplementary material for: Civil war and death in Yemen: Analysis of SMART survey and ACLED data, 2012–2019
Source: PLOS Glob Public Health. 2022 Aug 8;2(8):e0000581. doi: 10.1371/journal.pgph.0000581 (PMC10022117; doi:10.1371/journal.pgph.0000581)
Supplement: S3 Table — One-way ANOVA test to describe the association between four security levels and PCDR as calculated from 91 small-scale cluster surveys. Security levels were determined by generating security scores and insecurity indices. The insecurity indices were then classified into quantiles for analysis. (PDF) [file pgph.0000581.s004.pdf]

S3 Table: Association between security level and posterior crude death rate, Yemen, 2015 – 2019. *One-way ANOVA test to describe the association between four security levels and PCDR as calculated from 91 small-scale cluster surveys. Security levels were determined by generating security scores and insecurity indices. The insecurity indices were then classified into quantiles for analysis.*

	Degrees of freedom	Sum square	Mean Square	P-value
Security level (n=22)	3	0.02	0.01	0.41
Residuals	18	0.10	0.01	
