# Supplementary material for: Civil war and death in Yemen: Analysis of SMART survey and ACLED data, 2012–2019
Source: PLOS Glob Public Health. 2022 Aug 8;2(8):e0000581. doi: 10.1371/journal.pgph.0000581 (PMC10022117; doi:10.1371/journal.pgph.0000581)
Supplement: Attachment — Submitted filename: Reviewer and journal comments.docx [file pgph.0000581.s006.docx]

Journal Requirements:

1. Please update your Competing Interests statement. If you have no competing interests to declare, please state: “The authors have declared that no competing interests exist.”

This has been added.

2. Please ensure that you refer to Figure 2 in your text as, if accepted, production will need this reference to link the reader to the figure.

Figure 2 has been moved and renamed to be located where referenced in the text.

3. Please provide us with a direct link to the base layer of the map used in Figure 4, Figure 5, S1 Figure, and ensure this location is also included in the figure legend.

The link is provided for the map in the figure legend.

**Comments to the Author**

1. Does this manuscript meet PLOS Global Public Health’s [publication criteria](https://www.google.com/url?q=https://journals.plos.org/globalpublichealth/s/criteria-for-publication&source=gmail-imap&ust=1648524828000000&usg=AOvVaw04SVM8VgW-upjNRNR9DwYW)? Is the manuscript technically sound, and do the data support the conclusions? The manuscript must describe methodologically and ethically rigorous research with conclusions that are appropriately drawn based on the data presented.

Reviewer #1: Yes

Reviewer #2: Yes

2. Has the statistical analysis been performed appropriately and rigorously?

Reviewer #1: I don't know

Reviewer #2: I don't know

3. Have the authors made all data underlying the findings in their manuscript fully available (please refer to the Data Availability Statement at the start of the manuscript PDF file)?

The [PLOS Data policy](https://www.google.com/url?q=https://journals.plos.org/globalpublichealth/s/data-availability&source=gmail-imap&ust=1648524828000000&usg=AOvVaw1C49tAWzY80qaK5H1Yv0ds) requires authors to make all data underlying the findings described in their manuscript fully available without restriction, with rare exception. The data should be provided as part of the manuscript or its supporting information, or deposited to a public repository. For example, in addition to summary statistics, the data points behind means, medians and variance measures should be available. If there are restrictions on publicly sharing data—e.g., participant privacy or use of data from a third party—those must be specified.

Reviewer #1: No

Reviewer #2: No

4. Is the manuscript presented in an intelligible fashion and written in standard English?

PLOS Global Public Health does not copyedit accepted manuscripts, so the language in submitted articles must be clear, correct, and unambiguous. Any typographical or grammatical errors should be corrected at revision, so please note any specific errors here.

Reviewer #1: Yes

Reviewer #2: No

5. Review Comments to the Author

See below.

6. PLOS authors have the option to publish the peer review history of their article ([what does this mean?](https://www.google.com/url?q=https://journals.plos.org/globalpublichealth/s/editorial-and-peer-review-process%23loc-peer-review-history&source=gmail-imap&ust=1648524828000000&usg=AOvVaw1tYTGnnJnqWuF8bt1zszST)). If published, this will include your full peer review and any attached files.


**Do you want your identity to be public for this peer review?** If you choose “no”, your identity will remain anonymous but your review may still be made public.


For information about this choice, including consent withdrawal, please see our [Privacy Policy](https://www.google.com/url?q=https://www.plos.org/privacy-policy&source=gmail-imap&ust=1648524828000000&usg=AOvVaw3QPnolrqYy71XMkM4opTTw).

Reviewer #1: **Yes:** Sharif Ismail

Reviewer #2: **Yes:** Amy Hagopian

**Reviewer #1:**

Thank you for the opportunity to review this manuscript, which addresses an important set of questions relating to the impact of conflict on mortality rates in Yemen. This is an under-researched topic, and original health research on Yemen is in any case rare, so the paper is welcome. The results presented are important and the finding regarding the proportionate share of direct and indirect deaths, in particular, is counterintuitive. The caution shown by the authors in interpreting these results in the discussion – particularly by highlighting long running population vulnerabilities in Yemen that long pre-dated the conflict – is to be commended.

I provide some general comments here, and then more detailed comments by section further down.

**Reviewer #2:**

13 December 2021

Review by Amy Hagopian, PhD, University of Washington

Thank you for the opportunity to review the above-referenced paper. The study aims to estimate the death toll associated with the civil war in Yemen (2012-2019), using 91 household surveys, as well as data from ACLED (2015-2019). Researchers found a slight increase in the death rate, but most of the deaths were attributable to combat (as opposed to indirect deaths, a more common feature of modern conflict mortality estimates).

The paper is very interesting and worthy of publication. My review is organized by section, followed by some general and overall comments.

**REVIEWER COMMENTS ADDRESSED**

1. I would recommend a statistical review for aspects of the methodology in this paper – I am not qualified to comment on the appropriateness or construction of the Poisson-gamma model used to generate the main results.
2. Of the large team that contributed to this work, only one appears to be Yemeni and none are currently based in country (if the affiliation list is complete). Given that PLOS Global Public Health has set as its mission an effort to address entrenched global health inequalities, and to give voice to those often excluded, this imbalance in contributorship to the paper is somewhat disappointing and something to be addressed for future studies.

- Our Yemeni and on-the-ground colleagues are now properly acknowledged in the Acknowledgements section, but they remain unnamed to ensure their security.

3. An important issue in the construction of this analysis is the use of 2015 (the year in which the conflict was internationalised) as a cut-off point to distinguish BCDR from PCDR (as also indicated by table S1). The authors acknowledge this briefly in the discussion in line 373ff. While there was certainly a step-change in the intensity of conflict in that year resulting from international intervention, fighting had been ongoing internally since at least September 2014 (the point at which the Houthis entered Sanaa) and in some localised areas (especially in the North) intermittently but with some intensity to a point well before the beginning of the study time series in 2012. This may contribute to an overestimation of effects especially for governorates such as Hajjah and Al Jawf in the North where direct deaths in the pre-2015 period may have been significant, assuming that the BCDR does not account for violence-related mortality during the 2012-14 period. It may also help explain why the difference in pre-post CDRs is not that great. Do the authors have the data available from the earlier period to comment on this? What effect would incorporation of these numbers have on the TED estimate, in their view?

- This comment is very helpful for better articulating limitations and alternative explanations. The text has been revised to include the following: “We selected a time period for analysis based on available data and the formal recognition of the conflict in Yemen. However, there were appreciable levels of conflict throughout the country prior to the 2015 start date selected for the study.” (Lines 393-396)

4. Relatedly, treating the 2015-2019 period as a single block for PCDR calculation may mask large variations in conflict intensity over time. For example, ACLED data show large spikes in reported deaths in 2015 for civilians but markedly lower rates after this point, although this is admittedly not the case for combatants (<https://acleddata.com/2019/06/18/yemen-snapshots-2015-2019/>). The authors may wish to consider this as a possible explanation for the small different in pre-post CDRs observed in their study, in the discussion.

- The following text has been added to reflect this alternative explanation: “By analysing the post conflict period in the aggregate, we may have missed changes in mortality experience as the intensity of the conflict has changed over time. These potentially masked changes are an alternative explanation for the small differences in pre- and conflict crude death rates.” (Lines 396-398)

5. The authors rightly point out the surprisingly high overall contribution of direct mortality to the mortality rate. However, similar results have I think been documented elsewhere (albeit for more focused time periods) and some studies suggest CDRs as high as 1 per 10,000 person-days or more (e.g. <https://www.lshtm.ac.uk/south-sudan-full-report>). Are the authors able to draw any conclusions based on their data and reading of the wider literature as to how conflict intensity in Yemen compares with other settings?

- The findings from this study have been incorporated into the discussion of direct deaths to show that, while not commonplace, direct deaths in conflicts have substantially contributed to overall death rates. This gives more credence and contextualization to the findings of our study. The text now reads: “However, there are isolated occurrences where direct causes do constitute the majority of deaths, as seen in South Sudan from 2013-2018 [51]. In our study, Yemen is another rare example where almost two-thirds of excess deaths were the result of violent causes with the remaining third of deaths due to indirect causes.” (Lines 186 – 188)

6. Line 50-51: please provide the CIs around the BCDR and PCDR estimates here.

- Thank you for addressing this oversight – 95% Cis have been added.

7. Line 55: please spell out what PCDR is (you give this in the main text, but the abstract is the first time this abbreviation is seen by the reader).

- This has been corrected.

8. In some areas the phrasing is informal e.g., line 78 - “knee-deep in the largest cholera outbreak…” and could be amended without compromising the central messages that the scale and scope of conflict impact in Yemen has few contemporary precedents.

- Edits incorporated. (e.g., “knee-deep,” “fend for themselves”)

9. Line 88-99: this section reflects a general tendency to frame the conflict in Yemen as a proxy conflict between two distinct sides, identified in sectarian terms and by relationship to specific external actors. In fact, the conflict in Yemen has for some years been a multi-dimensional civil war, primarily driven by internal dynamics. Fighting between groups nominally opposed to the Houthis has at times been just as intense as between the Houthis and others. There is also an important secessionist dimension to the conflict in the South. International alliances for parties in the conflict have shown considerable fluidity over time. There are a number of sources to consider citing here from close observers of Yemen, including work by Bernard Haykel, Helen Lackner and Peter Salisbury.

- We thank the reviewer for this comment. A sentence has been added to better reflect the complexity of the conflict, and references have been added. The text now reads: “The Yemeni crisis features a complex network of both internal and external forces, with multiple layers of conflict – with distinct ideological, geopolitical, sectarian, and separatist interests – occurring simultaneously [9–11].”

10. Results – line 240ff: the authors have provided brief details of included surveys in table S2 but it would be helpful to have a clearer view of the content of these. Can the authors comment on the quality of included surveys? In addition, it would be helpful to have an indication of what the reported deaths rates in these individual surveys actually were over time, to (i) build a sense of the spread of the data (including outliers) and (ii) help address point 4 above.

- Thanks for this comment. We have added a brief summary of the information contained in SMART surveys.
- We included new text on lines 163 – 170 that indicated the mortality information contained in the survey reports and reference to supplementary data with information about the surveys and URL link as well.
- The following link supports Supplementary Table 2: <https://www.humanitarianresponse.info/en/operations/yemen/nutrition>.

**Reviewer #2: Please see comments in the attached file.**

***Abstract***

The last sentence of the 2^nd^ paragraph is quite confusing. By “/ its association” do authors mean the conflict period? Is this what you mean, perhaps: “To estimate pre-conflict and post-conflict mean death rates, we used a Poisson-Gamma model (useful for modeling situations involving continuous change) to analyze the distribution of deaths reported in the household survey over the study’s time period. Applying pre- and post-conflict death rates to total population numbers allowed us to estimate nationwide excess deaths, and test for association with security levels by governorate.”

- In lines 46 – 50 we corrected as follows: “We used a Poisson-Gamma model to estimate pre-conflict ($\mu_{p}$, baseline value) and conflict period ($\mu_{c}$) mean death rates using household survey data from 2012 – 2019. To analyse changes in the distribution of deaths and estimate nationwide excess deaths, we applied pre- and post-conflict death rates to total population numbers. Further, we tested for association between excess death and security levels by governorate.”

Note an unidentified acronym (PCDR).

- Corrected line 57 to read: posterior crude death rate.

You don’t say whether you think the .19 crude death rate is meaningfully different from a rate of .20, but here is where you might as well do that.

- Added “meaningfully” higher; we want to ensure that we do not allude to statistical significance. See line 55.

I assume Hajjah, Ibb and Al Jawf are governorates, but you don’t state that.

- Added “governorates” in line 58 for clarification.

***Introduction/background***

Your opening sentence is a bit a little more inflammatory than perhaps necessary; not because it’s not true, but because it’s better to show us than tell us. Which you then go on to do quite nicely.

- Edited to read: “The 2019 United Nations Office for Coordination of Humanitarian Affairs’ (UNOCHA) Humanitarian Needs Overview on Yemen describes the plight of a country that, despite the severity of the humanitarian crisis, is infrequently at the top of news headlines or global agendas.” See lines 67 – 70.

You might note that Yemen is the poorest country in the region, as that has implications later for using the SPHERE baseline average regional death rate.

- This has been incorporated into the text lines 100 - 102. It now reads: “Economically, Yemen – the poorest country in the Eastern Mediterranean World Health Organization (WHO) region – has largely collapsed.”

Your description of the conflict probably should more specifically call out the US and UK arms sales to Saudi Arabia, and the blockade of the ports that has inhibited food and aid imports. The missing end parentheses on line 98 (page 5) obscures meaning.

- This comment is well-noted. We have now incorporated it more explicitly into the text lines 93 - 95, which now reads: “The current Yemeni conflict became internationalized when Saudi Arabia and the Gulf states, with arms support from the United States and United Kingdom, began aiding the largely Sunni forces [9]. Concurrently, Iran has increasingly supported the Zaydi Houthi forces.”

It’s sort of odd to refer to a death count that may be inconsistent with your own findings that seems largely anecdotal (line 74, reference 5).

- This has been edited to highlight the mounting cost of the conflict without adding the seemingly anecdotal numbers. The text on lines 76 – 78 now reads: “By the end of 2017, health facility deaths and injuries directly attributable to conflict continued to mount [5].”

The sentence describing the GBD numbers (lines 85-87) are confusing. Perhaps better: *In 2016, the Global Burden of Disease study reported “conflict and terror” as the second cause of death overall and the leading cause of premature death in Yemen*. [7]

- This sentence has been edited for clarity (new lines 88 - 89) : “In 2016, the Global Burden of Disease Study (GBD) reported “conflict and terror” as the second cause of death and leading cause of premature death in Yemen [7].”

The sentence about the frustration among humanitarian workers seems unnecessary (and not directly related to your research question) unless you want to point us to a particularly good reference on that.

- This sentence has been removed.

The history of the conflict (at the end of the intro section) is important, but too long. Not all statements have supporting references.

- Superfluous text has been removed from the second paragraph of the introduction.

The last sentence (on examining the role of “armed violent incidents”) probably would be helpful much earlier, with a little hint at how you will do that. And maybe a better term is *conflict incidents involving weaponry, including air strikes*.

- We thank the editor for this comment. With the revision and edits of the introduction, we think that the structure should now be clearer. We also modified the text lines 120 – 122 for clarity.

I would make some other edits in the introduction section to reduce unnecessary words, offer more paragraph breaks, and enhance meaning, but I’ll leave that to the journal copy editor.

- Edits have been made to shorten sentences and break the text into more paragraphs.

***Methods***

Consider the reader who might like to replicate this work in a different conflict zone—the methods described are insufficient to replicate a similar approach.

- Thank you for raising this issue. We modified and included additional texts to properly capture the modeling process. See lines 182 - 192 and lines 208 - 210. To further assist the reader, see also supplementary document S1for the analysis process flow.

Also, many will read this paper for the methods, not just the results, so I’d like to see more details on the how the various data sets were assembled for collective analysis, and any “lessons learned” from those methods. For example, were the SMART criteria for inclusion of a survey sufficient, or do the researchers have any modifications to propose for future surveyors? (That would go to Discussion section)

- Thank you for raising this important point. We have added additional text (lines 169 – 173) and lines 262 – 264) for clarity on the assembling of the datasets. Our inclusion of surveys in the analysis is not dependent only on the SMART methodology but also on whether; the targeted population are Yemenis, if the survey collected mortality information and if the surveys reported CDR or sufficient information to estimate it. We included image of the selection criteria in Figure 3.

I’d like to see more hints in the first paragraphs about what measures were captured in these small-scale surveys.

- We included addition text (lines 147- 150) to reflect this.

We presume these are retrospective counts, so tell us the time periods. What subjects participated in these surveys? What kinds of variables were available? Figure 3 (untitled) is helpful, but the manuscript text could explain more of the process of selecting and using these surveys. For example, I take it you used ONLY surveys with mortality reports (N=97), but it would be helpful to know how these crude mortality reports were presented (counts for a specific geographic territory and time period)? You might even offer a list of the URLs to these surveys in a supplementary appendix for people who want to look at them.

- We included new text on lines 163 – 170 that indicated the mortality information contained in the survey reports and reference to supplementary data with information about the surveys and URL link as well.
- The following link supports Supplementary Table 2: <https://www.humanitarianresponse.info/en/operations/yemen/nutrition>.

Line 133: perhaps clearer: “we aggregate mortality statistics from 2864 individual geographical clusters in 91 small scale mortality surveys reported by six organizations”

- This suggestion is well received. The sentence now reads: “To circumvent the lack of official mortality data, we aggregated mortality statistics from 2,864 individual geographical clusters in 91 small scale mortality surveys collected by six organisations. All surveys are secondary, unidentified data sources.” (Lines 131-133)

The sentence in lines 146-149 is in passive voice, and trying to do too much; it’s confusing. Also, what is a “secured area” ?

- We agree that this sentence needs clarification. It has been revised to read: “Household surveys of death counts from areas with active hostilities from the Armed Conflict Location and Event Data Project (ACLED) and the Yemen Data Project (YDP) constitute the mortality data used in this study.”

Table 1: Perhaps clarify the “direct deaths” variable name to be “deaths from war violence”

- We have corrected it.

Mortality model: first paragraph, lines 163-166. Your mortality reports come from many small areas, right? Was simple aggregation sufficient? Did you have a count for each year? What areas did you miss, and how did you account for that?

- Thank you for pointing out this very important issue. Combining the estimates from the different zones/region in a specific year within a governorate gives us the mortality rate for that year. We used the estimated/reported yearly governorate level mortality rate to calculate the yearly number of deaths in the governorate as contained in our method. Lines 179 – 241. Given the available data the combination of estimates seems a reasonable approach. Most surveys in our analysis indicated that some areas were omitted die to insecurity, and we try to account for that by including mortality from ACLED in our final calculation and we acknowledge the limitations in our manuscript.

You will have readers who are not sophisticated in statistical methods, and you could make some efforts to be more accessible to them. PCDR is posterior crude death rate (named on page 10, line 165, although you use the acronym in the abstract, undefined). You might just note that the crude death rate is the number of deaths occurring among the population of a given geographical area during a given year, per 1,000 total population of that same area during the same year, unadjusted for the population’s age distribution.

- Thanks for this correction we have included additional text to this effect. See lines 167 – 169.

The lack of paragraph breaks merges the death information with the overall population estimate information—you should separate these topics to create clearer meaning.

- We have corrected it by creating a paragraph between lines 216 and 2018 to indicate the beginning of new information – the calculation of “excess deaths”.

The test of association between four security levels and PCDR in S3 is of great interest but buried in the supplement. Further, the table is very obscure. A caption could suggest how to interpret the findings. Briefly note in the Methods section what your goal was in making this calculation.

- We included few texts to illustrate the goal of this calculation. See lines 122 – 123 and 264 - 265.

Table 1 contents should not be right-justified.

- The text is now left-aligned.

***Results***

The information in the first paragraph about baselines is a little confusing. You start by telling us what is “often” done, and then why the rate from the SPHERE baseline sets a relatively low death rate comparison, and then tell us what you did (using a baseline from your own BCDR data), and then refer us to table 2. Should the SPHERE rate (.16) be in the table for reference purposes? In any case, Table 2’s rates should each have a footnote to the table stating the sources.

- Thanks for this point. We only included rates estimated from our in the table. We included data source in the footnote line 283.

Line 261 says “governorate-wise,” but I think you mean governorate-wide. In any case, do you need either word?

- We agree and we have corrected the text line 292.

The file labeled “S2 figure” has a title of S1.

- Corrected.

All titles of tables and figures should be able to stand alone, separate from the paper. For example, “S2 Table: Survey characteristics” should be “S2 – Characteristics of 91 small scale mortality surveys in Yemen reviewed for mortality estimates, 2012-2019.” All titles should have “Yemen” in them.

- All titles have been changed to better reflect the content of the figure/table. All titles now contain both “Yemen” and dates.

All tables and figures should have captions indicating the source of data, including the supplemental tables and figures. Figures 1, 2, 3, 4, 5, and 6 have no titles or captions at all.

- All titles have been changed to better reflect the content of the figure/table. All titles now contain both “Yemen” and dates.

Avoid acronyms in the table or figure titles. For example, S3 Table uses “PCDR”

- PCDR has been spelled out.

***Discussion***

It would help to break up the 2^nd^ paragraph, and provide a line of commentary after each concept. The first two sentences might be followed by a sentence reminding us that conditions before the current crisis weren’t that great either, hence perhaps post-conflict onset death tolls reflect that reality (you could lift the sentence from line 307). Then the sentence on deaths attributable directly to (not “due to”) armed violence would start a new paragraph. Tell us something a little more about that. And then finally, the sentence starting “Third” would be its own paragraph, and you already have a sentence explaining that.

- Additional text has been added to read: “Third, our index of human insecurity was not correlated with the estimated conflict period deaths, which were similar in all governorates regardless of their level of insecurity. The mere threat of air strikes and violence may have been enough to halt essential health care and other critical life-sustaining activities across the country, not just in governorates specifically targeted with intense air strikes and fighting.”

This sentence is constructed poorly (lines 343-344): *Armed violence, such as multiple target devices (e.g., bombs, grenades, or air strikes) or single target (snipers, killings) is a powerful force — not only on direct mortality but also on the pervasive disruption to civilian life*. Similarly, the sentence in the same paragraph at the top of page 19 is not structurally sound: *In Yemen, severe and widespread personal and community insecurity, both real and perceived, has become part of daily life and hence, a consequence to survival*. (Insecurity…has become…a consequence to survival??)

- This paragraph has been edited for clarity and brevity to now read: “Human insecurity in mainland governorates did not correlate positively with higher death rates, despite higher numbers of air strikes and armed attacks in some governorates. Armed violence has severe implications on direct mortality and pervasively disrupts civilian lives. Even when there are no direct hits, both multi-target (e.g., bombs, grenades, shells) and single-target (e.g., targeted killings, snipers) violence profoundly disrupt access to food, markets, healthcare, and other critical commodities by seeding fear of movements. Insecurity has become deeply embedded into the realities of daily life for Yemenis.”

I appreciate the discussion of indirect v. direct deaths. This sentence, though, is confusing owing to the use of passive voice: *Our analysis suggests that lower levels of indirect excess mortality may have been due to reduced deaths from active humanitarian aid, and a high baseline due to prolonged past conflic*t. (lines 358-359). Perhaps better: “Decades of active humanitarian aid to this poorest nation in the Middle East may have protected the population from expected conflict-related indirect deaths, and yet prolonged previous conflicts may also have served to elevate the baseline crude death rate.”

- This sentence has been changed to read: “The appreciable increase in humanitarian aid to Yemen following the formal, international recognition of the humanitarian crisis, in conjunction with the already high crude baseline death rates, may explain the observed reduction in indirect deaths.”

This sentence is important, but a little buried, “More than 50% of the surveys used in this study specifically reported excluding areas due to inaccessibility or insecurity.” It should be in the limitations section and perhaps even referred to earlier in the methods section as a caution.

- This has been noted in the methods section: “It is important to note that more than half of the surveys used reported excluding areas due to inaccessibility or insecurity.”
- This has also been reiterated in the methods section: “Predictably, this is reflected in surveys undertaken in Yemen, with more than 50% of surveys noting excluding areas due to insecurity or inaccessibility.”

And speaking of which, you need a paragraph that summarizes the many limitations of the study. Perhaps you can cut some sentences elsewhere and move them here.

- A limitations section has been explicitly added.

The discussion section contains a lot of content about fear, unpaid salaries, disruptions to daily life, and other things that were not a direct part of the study. Since these comments rely on data not collected for the study, they present opportunities to trim the length of the manuscript.

- Sections have been removed to better reflect the direct content of the study.

Line 394 and 395 need to be updated, unfortunately.

- These have been updated to better reflect the current geopolitical realities.

***In general***

- Lots of unnecessary passive voice that’s hard to follow and adds unnecessary words.
  - We have addressed.
- “Due to” is an accounting term. “Attributable to” is better.
  - This has been corrected.
- Check all acronyms for first-use definition. CRVS, for example.
  - Acronyms have been checked and full titles added where appropriate.
- Improve captions and titles for the figures and tables. Authors should assume these will be lifted out of context, and name the location and time frame in more detail, and interpret what the reader should be looking for.
  - Edits made.
- This manuscript could be tighter, and it needs another copy edit (which now I'm seeing PLOS won't provide, unfortunately).
  - Edits made.
- I did not review the statistical calculations, outside my area of expertise.
- The data really should be accessible to the reader, but were not in a supplemental file as far as I could tell.
  - This is now provided as supplementary material, but is not included for publication.
